# Comparative Evaluation of Dental Age vs. Chronological Age in Healthy and Underweight Children Aged 8-16 Years: A Cross-Sectional Study

**DOI:** 10.7759/cureus.79619

**Published:** 2025-02-25

**Authors:** Aarti S Bedia, Sumit V Bedia, Sayem A Mulla, Amit Patil

**Affiliations:** 1 Oral Medicine and Radiology, Bharati Vidyapeeth (Deemed to be University) Dental College and Hospital, Navi Mumbai, IND; 2 Prosthodontics, Bharati Vidyapeeth (Deemed to be University) Dental College and Hospital, Navi Mumbai, IND; 3 Dentistry, Bharati Vidyapeeth (Deemed to be University) Dental College and Hospital, Navi Mumbai, IND; 4 Conservative Dentistry and Endodontics, Bharati Vidyapeeth (Deemed to be University) Dental College and Hospital, Navi Mumbai, IND

**Keywords:** body mass index, calculated dental age, chronological age, dental age, underweight

## Abstract

Background

Tooth calcification is one of the most reliable methods of identification of dental maturation of an individual. When physical growth is concerned, body mass index (BMI) is the preferred method of assessment of growth and development. There exists a conjugation between the nutritional status of an individual and their growth including dental growth.

Aim

This study aimed to evaluate and compare the calculated dental age of healthy and underweight children belonging to the age group of eight to 16 years with their chronological age.

Materials and methods

After using appropriate sampling techniques, the study included a total of 120 children. The subjects ranged in age from eight to 16 years old, which matched their chronological age. There were 62 males and 58 females. The current study classified children as underweight or normal weight based on the WHO's recommended BMI for the Asian population. The subjects' mandibular teeth were measured using an orthopantomograph (OPG) and an intraoral periapical radiograph (IOPA), and tooth calcification was graded using the Demirjian method. All collected data underwent descriptive statistical tests first, followed by the Chi-square, Z, and Pearson's correlation coefficient tests.

Results

The calculated dental age was less than the chronological age in all the groups. No statistical difference was seen in chronological age in underweight and normal subjects. However, a statistically significant difference was noted for calculated dental age in underweight and normal subjects.

Conclusion and clinical significance

Calculated dental age can be used as a standard to assess dental development, especially in underweight individuals correlating to their BMI. However, chronological age alone cannot be used for age assessment although it can be used along with calculated dental age and BMI.

## Introduction

Physical growth and developmental manifestations serve as useful diagnostic criteria in dentistry. Dentists frequently use physical characteristics such as weight, height, skeletal maturation, and dental development to evaluate patients' growth and maturational status. These characteristics are subjected to biometric tests and compared to standards based on a large group of healthy subjects. Dental development schedules are used as indicators of growth and maturation in childhood because teeth develop and erupt in a predictable sequence and age range [1,2].

Serial radiographic studies of dentition are critical and useful methods for assessing intra-alveolar dental development throughout the various developmental stages of teeth. The radiographic evaluation of intra-alveolar growth and dentition calcification is a useful indicator of dental age and an index of a child's overall maturation [3].

Tooth development is a useful measure of maturity because it represents a series of identifiable events that occur in the same order from the start to a fixed endpoint. Dental developmental milestones can therefore be used to estimate age. Dental age is estimated using the rate of tooth buds' development and calcification as well as the progressive sequence of their eruption in the oral cavity [4]. Several methods have been developed to determine dental age based on the degree of calcification seen in permanent teeth. One such widely used method is provided by Demirjian, Goldstein, and Tanner. It includes distinct details based on shape criteria and root length proportion, with a focus on relative value to crown height rather than absolute length. Foreshortened or elongated projections of developing teeth will have no effect on the assessment's reliability [5].

Nutrition is an integral part when growth is concerned. Body mass index (BMI) is one of the most commonly used weights for height measurement. BMI has been recommended as the most acceptable measure of body fat in children and adolescents because it is both valid and reproducible as well as simple to use. It is frequently a routine parameter recorded to label an individual's nutritional and overall status as underweight, healthy, or obese. There is definitely a relation between chronological age, dental age, and BMI of a child [2,6-8]. The aim of this study was to evaluate and compare the calculated dental age between healthy and underweight children from eight to 16 years of age belonging to Wardha, Sawangi (Meghe) region.

## Materials and methods

The current cross-sectional study was carried out in the Department of Oral Medicine and Radiology of Sharad Pawar Dental College and Hospital in Sawangi (Meghe), Wardha, India. Prior to beginning the study, the attached institute's Institutional Ethical Committee approved it. The study started in the month of October 2008 and ended in the month of October 2009.

The sample size was determined using the formula for finite population. Keeping the confidence level at 75%, margin of error at 5%, and population proportion at 50%, a total of 120 children were included. Out of this, 60 control and 60 test groups were made. The subjects ranged in age from eight to 16 years old, which corresponded to their chronological age. There were 62 males and 58 females. To ensure proper subject selection, a thorough history of each subject was obtained as well as a general physical and oral examination. The current study used the WHO-recommended BMI for the Asian population to categorize children as underweight or normal weight because the mean or median BMI in the Asian population is lower than that in the non-Asian population [9]. The study only included subjects who met the World Health Organization's underweight and normal weight BMI criteria for Asians. In each case, guardians and subjects were asked to provide the exact date of birth of the subject under study. Subjects and their guardians provided written consent.

Subjects aged eight to 16 years old with Indian nationality and a known date of birth (DOB), clinically normal growth and development, no history of facial trauma or injury, and a calculated BMI of less than 25 kg/m^2^ were eligible. Patients with a known history of a congenital anomaly; developmental and/or systemic disorders; as well as a long illness history, congenitally missing, impacted, or transposed teeth; or extraction of any permanent teeth were excluded, as was anyone with a calculated BMI greater than 25 kg/m^2^.

Subjects with BMIs ranging from 18.5 to 24.9 kg/m2 were included in the control group, while those with BMIs less than 18.5 kg/m^2^ were considered in the study group. The subjects' mandibular teeth were recorded with an orthopantomograph (OPG) and an intraoral periapical radiograph (IOPA), which were processed before being interpreted. Tooth calcification was graded using the method described by Demirjian et al., in which permanent mandibular central and lateral incisors, canines, premolars, and permanent first and second molars of the left side were assigned one of eight calcification stages, A to H [5].

The subject's chronological age was calculated by subtracting their birth date from the date the radiograph was taken. It was expressed in terms of years and months. The dental age was determined using the Demirjian method. The panoramic radiograph revealed seven teeth on the left side of the mandible. The development stage of each tooth was scored using the conversion table described by Demirjian et al. The scores of all seven teeth were added together to produce a total maturity score, which was then converted to dental age using the table provided by the same authors, resulting in the calculated dental age [5].

All collected data was compiled in MS Excel (Microsoft Corporation, Redmond, Washington, United States) and statistically tested. All collected data was subjected to descriptive statistical tests first, followed by the Chi-square, Z, and Pearson's correlation coefficient tests. For all statistical tests, p < 0.05 was regarded as statistically significant. The IBM SPSS Statistics for Windows, Version 26 (Released 2019; IBM Corp., Armonk, New York, United States) was used to statistically analyze the data.

## Results

The present study included 120 children (60 in the control group and 60 in the study group) aged eight to 16 years old. Table 1 shows the dental age, chronological age, weight, height, and BMI distribution of the total subjects.

**Table 1:** Dental age, chronological age, weight, height, and BMI distribution of the total subjects (120 participants) BMI: body mass index

Variables	Range	Mean ± SD
Dental age	7.70-16.00 years	12.16 ± 2.16
Chronological age	8.01-15.02 years	12.22 ± 1.98
Weight	24-52.30 Kg	34.87 ± 5.56
Height	1.20-1.62 meters	1.37 ± 0.09
BMI	16.05-22.84 Kg/m^2^	18.24 ± 1.70

The statistical significance of the calculated dental age comparison in underweight and normal subjects was significant in males (0.01, p < 0.05), females (0.008, p < 0.05), as well as the total subjects (0.00, p < 0.05), which indicate that there is a significant difference in calculated dental age in underweight and normal subjects and that standard calculated dental age can be used to assess dental development in relation to BMI (Table 2).

**Table 2:** Comparison of mean calculated dental age in normal and underweight subjects BMI: body mass index The statistical test used was the Chi-square test

	Normal	Underweight	z-value	p-value
Total subjects (120)	12.84 ± 1.94 years (mean BMI = 19.72)	11.47 ± 2.17 years (mean BMI = 16.73)	3.62	0.000 S, p < 0.05
Male subjects (58)	12.50 ± 1.90 years (mean BMI = 19.76)	11.17 ± 2.25 years (mean BMI = 16.70)	2.50	0.01 S, p < 0.05
Female subjects (62)	13.24 ± 1.95 years (mean BMI = 19.67)	11.78 ± 2.09 years (mean BMI = 16.76)	2.74	0.008 S, p < 0.05

The correlation between the chronological age and calculated dental age in male, female, and total subjects was statistically significant. Thus, the calculated dental age is significantly different from the chronological age in male, female, and total subjects but with a positive correlation (Table 3).

**Table 3:** Correlation of the chronological age and calculated dental age in male, female, and total subjects Statistical test used was Pearson's correlation coefficient test

Parameters	Chronological age (years)	Calculated dental age (years)	n	Correlation coefficient r-value	p-value
Total subjects	12.22 ± 1.98	12.16 ± 2.16	120	0.960	0.000 S, p < 0.05
Male subjects	11.94 ± 2.01	11.85 ± 2.17	62	0.960	0.000 S, p < 0.05
Female subjects	12.53 ± 1.92	12.48 ± 2.13	58	0.955	0.000 S, p < 0.05

## Discussion

Radiographic evaluation of dental development on the basis of calcification serves as an excellent tool for the evaluation of the dental age of an individual. It also helps in understanding the growth and development status of the concerned person. This along with BMI has the potential to diagnose a number of malnutrition or obesity cases at an early stage. Dentists along with physicians play an essential role in this procedure. The present study was done to evaluate and compare hand-in-hand the calculated dental age with chronological age in healthy and underweight children aged eight to 16 years.

Although there are various methods for determining age, a universal system has not been developed due to the differences between ethnic populations. In the current study, the teeth calcification stages were studied using Demirjian's method, which is applicable to the Indian population, is considered highly accurate, and is one of the most commonly used systems [10].

Differences in development among children of the same chronological age have given rise to the concept of physiological age as a means of defining progress toward complete development or maturity in the individual child. Thus, physiologic age and developmental age are used to describe the status of a specific child. The chronologic or calendar age provides only a rough estimate of the range of development observed at any given age. The dental system is an essential component of the human body; its growth and development can and should be investigated alongside other physiological maturity indicators such as bone age, menarche, height, and weight. However, a radiological examination of tooth development and mineralization provides significantly more information and has been shown to be consistent with chronological age [11].

A statistically significant correlation was found between chronologic age and calculated dental age in male, female, and total subjects (r = 0.960, r = 0.955, and r = 0.960, respectively). The calculated dental age was less than the chronologic age in all groups, including males, females, and total subjects. Prabhakar et al. discovered a highly significant correlation between chronologic age and obtained dental age (r = 0.94 in males, r = 0.95 in females), which is consistent with the results of the current study [12]. Similarly, Hägg and Matsson discovered a high correlation (r = 0.7 and 0.9) between chronologic age and calculated dental age in children aged 3.5 to 6.5 years, regardless of the method used [13], and Gulati et al. also reported a statistically significant correlation (r = 0.86) [14].

In the present study, there existed a significant difference between the chronologic age and calculated dental age in males which was similar to studies done by Kumar et al. [2], Vallejo-Bolanos et al. [15], Green [8], Macha et al. [16], as well as Hegde and Sood [11]. Factors such as ethnic background, nutritional status, etc., are thought to be responsible for the difference between these parameters [16]. There is no statistically significant difference in chronological age in underweight and normal subjects; hence, only chronological age cannot be used to assess dental development in relation to BMI. This hypothesis is also supported by Panchbhai et al. [17] who stated that it is just an arbitrary measurement.

The relationship between BMI and dental age difference in total underweight subjects was not found to be significant. The mean dental age difference in all underweight subjects was found to be negative, indicating delayed dental development. The correlation between BMI and the dental age difference was found to be significant in all normal subjects, indicating that as BMI decreases, the dental age difference increases significantly. A positive mean dental age difference indicates that dental development accelerates as BMI rises in healthy individuals. The correlation between BMI and dental age difference in total subjects was significantly positive, indicating that as BMI decreases from normal to underweight, the dental age difference decreases, implying that development is delayed. It is possible that inadequate nutrition is one of the culprits behind delayed dental development.

The findings of this study highlight the importance of dental practitioners providing regular BMI screenings, which can be done simply by recording the height and weight of all children and calculating BMI status. This would aid in the early detection of BMI changes and subsequent intervention, as low BMI may be a contributing factor to delayed dental development. In determining the dental age of children, the calculated dental age must be considered in addition to the chronologic age. In normal and underweight children, the chronologic age is significantly higher than the calculated dental age. The calculated dental age of normal and underweight children differed significantly, whereas the chronologic age of normal and underweight children did not differ significantly. As a result, the calculated dental age can be used to determine the dental age of a person whose birth date is unknown. As healthcare providers, we should provide nutritional education not only to promote oral health but also to maintain overall health. Such simple measures may raise awareness and reduce the long-term health consequences of childhood underweight. As part of forensic dentistry, dental age estimates can be performed to evaluate the phases of tooth eruption and growth, frequently using X-rays. Techniques such as the Ubelaker and Demirjian techniques compare reference data with observed phases. Tooth development is influenced by a number of factors, including environment, diet, and genetics. When records are absent or there are unidentifiable remains, they might be utilized to approximate age.

Limitations and future recommendations

The relationship between tooth maturity and other developmental parameters varies according to race [18]. When generalizing this finding to other racial groups, exercise caution. This study's limitations include its cross-sectional design and the small number of subjects drawn from a single location. More research with a large number of samples is needed to compile a comprehensive table of dental age conversion from maturity score. Research is also needed to develop new scoring measures for Indian children. Furthermore, the number of subjects was severely limited when separated by age, gender, and BMI status. Furthermore, the subjects included had a very narrow BMI range of 16.5 to 22.84. More studies are needed with more samples in each category of BMI recommended by the WHO for Asian populations, from severely underweight (BMI < 16kg/m^2^) to obese class III (BMI > 40 kg/m^2^), to effectively observe the effect of BMI on dental development. Future longitudinal studies can be beneficial compared to cross-sectional studies.

## Conclusions

Calculated dental age was less than chronological age in the present study which indicated that it can be taken into consideration along with chronological age for the evaluation of the age of an individual. However, chronological age alone cannot be used to assess dental development when BMI is also concerned. Calculated dental age can be used to assess dental development in conjugation with the BMI of an individual. It can also be used to evaluate the dental age of a subject with an unknown date of birth.
